# Maternal Depressive Symptoms and Their Association with Breastfeeding and Child Weight Outcomes

**DOI:** 10.3390/children8030233

**Published:** 2021-03-17

**Authors:** María Pineros-Leano, Jaclyn A. Saltzman, Janet M. Liechty, Salma Musaad, Liliana Aguayo

**Affiliations:** 1 School of Social Work, Boston College, 140 Commonwealth Avenue, Chestnut Hill, MA 02457, USA; 2 Illinois Transdisciplinary Obesity Prevention Program, University of Illinois Urbana-Champaign, 905 S. Goodwin Ave., Urbana, IL 61801, USA; jaclyn.saltzman@gmail.com (J.A.S.); jliechty@illinois.edu (J.M.L.); salma.musaad@bcm.edu (S.M.); liliana.aguayo-markes@emory.edu (L.A.); 3 Human Development and Family Studies, University of Illinois Urbana-Champaign, 905 S. Goodwin Ave., Urbana, IL 61801, USA; 4 School of Social Work, University of Illinois Urbana-Champaign, 1010 W. Nevada St., Urbana, IL 61801, USA; 5 Carle Illinois College of Medicine, University of Illinois Urbana-Champaign, 807 South Wright Street, Champaign, IL 61820, USA; 6 USDA/ARS Children’s Nutrition Research Center, Department of Pediatrics, Baylor College of Medicine, 1100 Bates Street, Houston, TX 77030, USA; 7 Hubert Department of Global Health, Emory University, 1518 Clifton Rd. NE, Atlanta, GA 30322, USA

**Keywords:** pediatric obesity, maternal depression, breastfeeding

## Abstract

Children of mothers with depressive symptoms are at a higher risk for psychosocial, behavioral, and developmental problems. However, the effects of maternal depression on children’s physical growth are not well understood. To address the gaps in the literature, this study examined the association between maternal depressive symptoms, breastfeeding behaviors, and child weight outcomes. Data from 204 mother–child dyads who participated in the STRONG Kids 1 Study were used. Mothers and children were assessed twice when the children were 3 and 4 years old. Height and weight measurements of children and mothers were collected by trained researchers during both assessments. Multiple linear regression and analysis of covariance tests were used to examine the associations between maternal depressive symptoms, breastfeeding, and age and sex-adjusted child body mass index percentile. Recurrent maternal depressive symptoms when the child was 3 and 4 years old were not associated with child body mass index percentiles (BMI-P) at age 4. Mothers who breastfed for at least 6 months had significantly lower depressive symptoms when their children were 3 years of age, but the differences did not persist at age 4. In this community sample, maternal depressive symptoms were not associated with child BMI-P, regardless of breastfeeding duration.

## 1. Introduction

Children of mothers with depressive symptoms are at higher risk for psychosocial, behavioral, and developmental problems [1,2,3,4,5]. However, the effects of maternal depression on children’s physical growth are not well understood. Systematic reviews examining the association between maternal depression and childhood weight have reported mixed findings. This is in part due to the paucity of prospective studies investigating the association and because of the limited evidence investigating the impact of duration and chronicity of maternal depressive symptoms on child weight [6,7]. In a systematic review, the authors identified nine prospective studies investigating associations of episodic and chronic depression with child weight [6]. The results suggest that episodic depression is not associated with risk for childhood obesity [6]. Conversely, chronic depression was associated with greater risk for childhood overweight [6]. Recent studies suggest similar trends [8,9]. A study of 332 Latino children found that children of mothers with chronic depressive symptoms were more than twice as likely to be overweight/obese at age 7. The study also found that episodic depressive symptoms in mothers did not increased the likelihood of child overweight/obesity at age 7 [8].

One potential pathway through which maternal depression may influence children’s physical growth is by influencing the likelihood and duration of breastfeeding [10,11,12]. There is strong evidence in the literature documenting an association between maternal depression and decreased likelihood of breastfeeding initiation and duration [10,11]. Breastfeeding, in turn, is associated with healthier child weight outcomes [13,14,15]. However, only a handful of studies have investigated the interplay between maternal depression, breastfeeding, and child weight outcomes.

In the United States, the highest incidence of obesity occurs before the age of 5 years [16]. However, to our knowledge, no study has examined the contributions of breastfeeding in the association between maternal depression and child weight outcomes among children between the ages of 2 and 5 years. To address this gap, this study examines the association of maternal depressive symptoms and breastfeeding with childhood weight outcomes. Three hypotheses were developed based on prior literature and data availability: First, we hypothesized that children whose mothers reported recurrent depressive symptoms when children were age 3 and age 4 would have higher body mass index percentiles (BMI-P) at age 4 than children whose mothers did not report depressive symptoms. Second, we expected that breastfeeding for 6 months or more would be associated with lower child BMI-P at age 4. Lastly, we hypothesized that breastfeeding for 6 months or more would be associated with lower maternal depressive symptoms when the child was 3 and 4 years of age.

## 2. Methods

### 2.1. Sample and Participants

This study used secondary data from the STRONG Kids program, a three-wave prospective panel study of parent–child dyads (*n* = 496) that investigated the determinants of childhood obesity among 2 to 5-year-old children. Participants were recruited from preschools in central Illinois. Preschools were selected using three criteria: (1) preschools had to be registered with the Bureau of Child Care and Development, (2) preschools had to be within a radius of 65 miles from the study center, and (3) each preschool had to have at least 24 children enrolled in the age range (children aged 2–4 years). Out of 33 eligible preschools, 30 agreed to participate. Participation rate by parent–child dyads at each preschool ranged from 60 to 95%. Parents were invited to participate in the study, and enrolment started at the beginning of 2009. The inclusion criteria were parents/caregivers who (1) had children between the ages of 2 and 4 and (2) attended one of the participating preschools. Parents filled out questionnaires online or in paper-and-pencil format (for study details, see Harrison and Liechty, 2012) [17].

For the present study, we used data from Wave 1, collected when the children were 3 years old (mean = 3.09 years old, *sd* = 0.6 years) and wave 2, when the children were 4 years old (mean = 4.3 years old; *sd* = 0.62 years). Parent–child dyads were excluded based on the following criteria: (1) they did not participate in Wave 1 and Wave 2, (2) the parent was not a biological mother (which precludes breastfeeding), (3) parent did not speak English or Spanish, and (4) the child did not have complete BMI-P measurements. The analytical sample was comprised of 204 mother–child dyads.

### 2.2. Measures

Maternal Depressive Symptoms. Maternal depressive symptoms were assessed once at each Wave—1 and 2—using the mother’s self-report on the depression subscale of the Depression, Anxiety, and Stress Scale (DASS-21) [18]. The DASS-21 scale has 21 items, and the depression subscale consists of seven items. On the seven-item depression subscale, mothers reported the degree to which they experienced a variety of depressive symptoms over the past week. Responses ranged from 0 (never) to 3 (almost always). Depressive symptoms were modelled continuously and categorically. When modelled continuously, higher scores indicate more depressive symptoms (range = 0 to 21), and the scale demonstrated adequate reliability in this sample (α = 0.85) [18]. When maternal depressive symptoms were modelled categorically, we used a score of ≥10 to indicate mild to severe depressive symptoms, following recommendations for the instrument. Participants with a score of ≥10 during either Wave 1 or 2 were considered to have episodic depressive symptoms, and scores ≥10 at both Waves 1 and 2 were coded as having recurrent depressive symptoms.

Breastfeeding. Breastfeeding was measured at Wave 1 using a screener designed to collect children’s dietary history and measure breastfeeding initiation and duration. Mothers indicated whether they breastfed their child at eight different time periods in the first two years of life: <1 month, 2–3 months, 4–5 months, 6–9 months, 10–12 months, 13–18 months, 19–24 months, and >24 months. The question asked mothers whether they were breastfeeding at all, rather than whether they were exclusively breastfeeding. A dichotomous variable was created to describe infants who were breastfed for less than 6 months and children who were breastfed for 6 months or more. The cutoff of 6 months was chosen in line with current recommendations from the Center for Disease Control and Prevention (CDC), the American Academy of Pediatrics, and the World Health Organization [19].

Child body mass index percentile. Child BMI-P was calculated from child height and weight measurements. Child height was measured by trained researchers using a stadiometer (Seca, Model 242, Hanover, MD, USA), and weight was obtained using a digital scale (HealthOmeter, Model 349KLX, Boca Raton, FL, USA) at Waves 1 and 2. The CDC growth charts and recommendations were used to calculate age- and sex-adjusted BMI-P and obesity (age- and sex-adjusted BMI-P >95th) [20].

Covariates. Mothers self-reported age, income, race/ethnicity, education, and participation in the Women Infant and Children (WIC) program at Wave 1, and their BMI measurements in kg/m^2^ at Waves 1 and 2 (calculated from height and weight measurements collected by trained researchers) were examined as potential covariates. Preliminary correlation analysis indicated that child BMI-P at Wave 1 was correlated with maternal BMI at Wave 1, and child BMI-P at Wave 2 was correlated with maternal BMI at Waves 1 and 2. 

### 2.3. Analytic Strategy

Descriptive and summary statistics were examined to evaluate whether data conformed to distribution assumptions for multiple linear regression and analysis of covariance (ANCOVA) tests. The association between recurrent depressive symptoms and BMI-P at Wave 2 was examined using multiple linear regression. We first tested an unadjusted model and then a model adjusted for change in child BMI-percentile from Wave 1 to Wave 2 and for maternal BMI at Wave 2. The association between breastfeeding duration and child BMI-P at Wave 2 was tested using multiple linear regression. Lastly, the association between maternal depressive symptoms and breastfeeding duration was tested using ANCOVA. We initially tested an unadjusted model and then a model adjusted for maternal BMI at Wave 2. All analyses were conducted using SPSS, Version 25 (SPSS, Chicago, IL, USA).

## 3. Results

A total of 204 mother–children dyads were included in the study (see Table 1 for sample characteristics). The majority of mothers (mean age = 32) were White (73.5%), and most had at least a college education (72.4%). Most mothers initiated breastfeeding (85.8%), and almost half (49%) of the sample breastfed their children for 6 months or more. The prevalence of elevated depressive symptoms at either Wave 1 or Wave 2 was 13.2%. Recurrent elevated depressive symptoms at Wave 1 and again at Wave 2 were reported in 5.9% of mothers. At Wave 1, 16% of children were overweight, and 7% were obese. By Wave 2, 18% of children were overweight, and nearly 10% were obese.

Based on linear regression analysis, there were no associations between recurrent maternal depressive symptoms (at Waves 1 and 2) and child BMI-P at Wave 2 in unadjusted or adjusted models (β = 0.02; CI = [−12.24, 17.32]; Table 2). Breastfeeding for 6 months or more was associated with lower child BMI-P at Wave 2 in the unadjusted model, but this was not sustained after adjusting for maternal BMI and child BMI-P change from Wave 1 to Wave 2.

Finally, after adjusting for maternal BMI at Wave 2, mothers who breastfeed for 6 months or more had significantly lower depressive symptom scores at Wave 1 (F = 4.03; *p* = 0.04; Table 3). Breastfeeding for 6 months or more was associated with depressive symptoms at Wave 2 only in the unadjusted model (results not shown).

## 4. Discussion

This study examined the influence of recurrent maternal depressive symptoms on children’s growth during a critical period of childhood development [21]. The results revealed that recurrent maternal depressive symptoms when children were 3 and 4 years old were not associated with children’s BMI-P at age 4. Breastfeeding for 6 months or more was not associated with lower BMI-P at age 4, after controlling for covariates. Finally, this study showed that breastfeeding for 6 months or more was significantly associated with lower maternal depressive symptoms when the child was 3 years of age. Overall, these results indicate that the relationship between maternal depressive symptoms and children’s weight is rather complex.

The unexpected results from this study can be a consequence of several factors, including a small sample size or the focus on reoccurrence rather than chronicity. It is also possible that maternal depressive symptoms may not be associated with healthier child weight. Previous longitudinal studies have found similar results. For instance, in a recent longitudinal study of 1130 mother–children dyads, researchers found that higher maternal depressive symptoms when the child was two months old were associated with lower child BMI z-scores at age 6, but maternal depressive symptoms when the child was 6 years old were not associated with child BMI z-score at age 6 [22]. Another longitudinal study of Latino mother–child dyads (*n* = 118) found that maternal depressive symptomatology over time was not associated with child obesity at age 9. Instead, higher maternal depressive symptoms during the prenatal period, when the child was 6 months old and when the child was 5 years old, were associated with lower likelihood of chronic child obesity, defined as having obesity at ages 5 and 9 [23]. Similarly, a longitudinal study of 3792 mother–children dyads from Pelotas, Brazil found that maternal depressive symptoms measured when the children were 12, 24, and 48 months old were not associated with child weight at 48 months [24]. Another longitudinal study of 3500 mother–child dyads found that maternal depression during early childhood was not associated with child obesity development over time [25]. Taken together, these findings indicate that there are several factors that need to be taken into account to better understand the relationship between maternal depressive symptomatology and child weight over time. For instance, it is possible that clinical depression, but not depressive symptomatology, has an effect on childhood obesity development through feeding choices, decreased emotional regulation, or other social determinants of health. It is also possible that more severe depressive symptoms during the first 1000 days have long-lasting consequences that impact children in several ways, including child weight [7]. This intricate and complex potential relationship between maternal depressive symptoms and child weight need to be better understood. More studies are necessary to determine whether maternal depressive symptoms are associated with child weight and exactly under which conditions.

Another important finding from this study was that breastfeeding for 6 months was associated with lower maternal depressive symptoms when the child was 3 years old, but not when the child was 4 years old. Previous studies suggest there is a bidirectional relationship between maternal depressive symptoms and breastfeeding initiation and duration [10,11,12]. Ours is not the first study to document an association between breastfeeding and maternal depression. In a systematic review, researchers found that out of 49 studies, most studies confirmed the association of breastfeeding with lower maternal depressive symptoms [26]. However, studies examining the long-term effects of breastfeeding for 6 months or more on maternal depressive symptoms, to which we can compare our findings, are lacking. Investigating the effects that breastfeeding has on maternal depressive symptomatology is warranted. It is particularly important to understand breastfeeding perceptions and behaviors in diverse populations because there are well-documented breastfeeding racial/ethnic disparities. Identifying the conditions under which a relationship can be observed between breastfeeding and maternal depressive symptoms could play an important role in mothers’ decision-making around breastfeeding and have important clinical implications. Despite the paucity of research in this area, it is important to promote breastfeeding whenever possible and to provide the necessary means to ensure that mothers who want to breastfeed have sufficient support to do so. Breastfeeding promotion is an important area where healthcare providers can intervene by providing relevant facts about its importance and by referring mothers to specialists who can help promote breastfeeding.

This study is not without limitations. Given the sample size and sample characteristics (with a majority of highly educated and white women), our results are not generalizable to other populations. We did not have information available on maternal depression during the perinatal period; therefore, we were unable to control for it in the statistical models. Further, we were not able to stratify results by severity of depressive symptoms (i.e., mild, moderate, severe), socioeconomic status, or race/ethnicity. Future longitudinal studies need to examine the impact of depressive symptom severity, timing of depression, and demographic factors on the association between maternal depressive symptoms, breastfeeding, and child obesity risk.

## 5. Conclusions

Considering the high incidence of childhood obesity in the United States, it is necessary to investigate potential behavioral and mental health factors that can influence its development during early ages [16]. Maternal depression was not associated with child weight outcomes in this relatively homogenous sample of families, which aligns with some—but not all—extant research. Further investigation of a) the long-term association between maternal severe and chronic depressive symptoms and child weight outcomes and b) the moderating and mediating effects of social determinants of health is needed. For instance, future studies should aim to examine the association between chronic maternal depression and childhood obesity among women of color with and without access to health insurance. A better understanding of the conditions that influence the associations between maternal depressive symptoms, breastfeeding, and child weight gain can help providers and researchers develop effective interventions to prevent maternal depression and childhood obesity.

## Figures and Tables

**Table 1:** Demographic Characteristics of Participants (*n* = 204).

Characteristic	*n*	%	M	SD
**Maternal Race/Ethnicity**				
White	150	73.50		
Black	20	9.80		
Latino/a	12	5.90		
Asian	18	8.80		
Other	3	1.50		
Missing	1	0.50		
**College graduate**	147	72.40		
**Marital status**				
Single	27	13.20		
Married	160	78.40		
Separated	3	1.50		
Widowed	1	0.50		
Cohabiting	10	4.90		
Missing	3	1.50		
**Income**				
<$25,000	29	14.2		
$25,000–$39,999	19	9.3		
$40,000–$69,999	46	22.5		
$70,000–$99,999	52	25.5		
>$100,000	48	23.5		
Missing	10	4.9		
**Maternal BMI category W1**				
Underweight (BMI < 18.5 kg/m^2^)	4	2.00		
Healthy weight (BMI 18.5–24.9 kg/m^2^)	103	50.00		
Overweight (BMI 18.5–24.9 kg/m^2^)	41	20.10		
Obesity (BMI < 18.5 kg/m^2^)	46	22.50		
Missing	10	4.90		
**Maternal BMI category W2**				
Underweight (BMI < 18.5 kg/m^2^)	5	2.50		
Healthy weight (BMI 18.5–24.9 kg/m^2^)	96	47.10		
Overweight (BMI 25.0–29.9 kg/m^2^)	48	23.50		
Obesity (BMI ≥ 30.0 kg/m^2^)	51	25.00		
Missing	4	2.00		
**Breastfeeding**				
Never	29	14.20		
Less than 6 months	75	36.80		
6 months or more	100	49.00		
**Maternal depressive symptoms**				
No depressive symptoms	143	70.10		
Episodic depressive symptoms	27	13.20		
Recurrent depressive symptoms	12	5.90		
Missing	22	10.80		
**Maternal depressive symptoms at Wave 1**	199		2.18	1.79
**Maternal depressive symptoms at Wave 2**	187		2.10	2.83
**Maternal age (years)**			32.44	5.19
**Child sex**				
Female	112	54.90		
**Child age at W1 (years)**			3.09	0.60
**Child age at W2 (years)**			4.30	0.62
**Child BMI percentile W1**			61.62	25.91
**Child BMI percentile W2**			62.80	28.07

Note. BMI = body mass index, M = mean, SD = standard deviation. Bold means it’s a variable.

**Table 2:** Linear regression analysis of the association between recurrent maternal depressive symptoms from Wave 1 and Wave 2 and child BMI percentile at Wave 2.

	Model 1				Model 2			
Variables	β	Unst. B	SE	95%CI	β	Unst. B	SE	95%CI
Recurrent maternal depressive symptoms	0.04	4.99	8.49	−11.77, 21.75	0.02	2.54	7.49	−12.24, 17.32
Change in child BMI-P from Wave 1 to Wave 2					0.45 *	0.70	0.10	0.50, 0.90
Maternal BMI at Wave 2					0.22 *	0.88	0.29	0.35, 1.41

Note. * *p* < 0.01. BMI = body mass index; BMI-P = BMI percentile; Unst. B = Unstandardized B coefficient; SE = Standard Error; 95%CI = 95% Confidence Interval. Model 1 was unadjusted. Model 2 adjusted for change in child BMI percentile from Wave 1 to Wave 2 and for maternal BMI at Wave 2.

**Table 3:** Analysis of covariance of breastfeeding for 6 months or more and maternal depressive symptoms from Wave 1.

	Model 1					Model 2				
Variables	df	SS	MS	F	*p*	df	SS	MS	F	*p*
Breastfeeding for 6 months or more	1	47.74	47.74	6.28	0.01	1	28.65	28.65	4.03	0.04
Maternal BMI at Wave 2						3	19.74	6.58	0.92	0.43

Note. *p* < 0.01. BMI = body mass index. Model 1 was unadjusted. Model 2 adjusted for maternal BMI at Wave 2.

## Data Availability

Data for this study are available under contractual agreement. Data can be requested from the first author with permission from the STRONG Kids 1 Team.
